# A social justice perspective on access to human rabies vaccines

**DOI:** 10.1016/j.vaccine.2019.01.065

**Published:** 2019-03-27

**Authors:** Diorbhail Wentworth, Katie Hampson, Samuel M. Thumbi, Athman Mwatondo, Gati Wambura, Nai Rui Chng

**Affiliations:** a Institute of Health & Wellbeing, College of Social Sciences, University of Glasgow, Glasgow G12 8QQ UK; b Institute of Biodiversity, Animal Health & Comparative Medicine, University of Glasgow, Glasgow G12 8QQ UK; c Center for Global Health Research, Kenya Medical Institute of Research, Kisumu, Kenya; d Washington State University – Global Health Program, Nairobi, Kenya; e Paul G. Allen School for Global Animal Health, Washington State University, Pullman, USA; f Zoonotic Disease Unit, Ministry of Health and Ministry of Agriculture, Livestock and Fisheries, Nairobi, Kenya

## Abstract

Rabies kills tens of thousands of people every year despite being entirely vaccine preventable. Key global health actors have launched a country-driven plan to achieve zero human deaths from dog-mediated rabies by 2030 worldwide. This partnership has recently been strengthened by Gavi, the Vaccine Alliance’s decision to invest in human rabies vaccines for post-exposure prophylaxis (PEP). While nation states are key to rabies elimination, the importance of Gavi’s role cannot be understated. Unlike any other actor, Gavi can directly address an otherwise intractable market failure in the inadequate supply of rabies PEP. In this commentary, we employ the Capabilities Approach to identify the barriers to PEP access that lead to this market failure and, as a result, unnecessary deaths and suffering. We show the role that Gavi can play in reducing exposure of PEP supply to market forces as a matter of social justice, and hence redress the inequity underlying human rabies deaths.

Canine rabies kills tens of thousands of people every year despite being a disease that is entirely vaccine preventable, and one for which effective vaccines have existed for over a century [1]. Mass dog vaccination has led to the elimination of rabies spread by domestic dogs from high-income countries. Rabies now remains as a disease of impoverished communities in low- and middle-income countries (LMICs). More than 95% of deaths occur in Africa and Asia, and mostly among people living in rural, underserved populations where dog vaccination is rare [2]. Following a rabid dog bite, prompt post-exposure prophylaxis (PEP) is the only way to ensure the invariably fatal onset of rabies is prevented [3]. Modelling suggests that without scaled up dog vaccination and under current PEP access, over 1 million people will die of dog-mediated rabies between 2020 and 2035 [4]. Mass dog vaccination is essential for the elimination of canine rabies, but here we address the role of PEP provision.

In June 2018, the World Health Organization (WHO), the World Organisation for Animal Health (OIE), the Food and Agriculture Organization of the United Nations (FAO) and the Global Alliance for Rabies Control (GARC) came together to launch a country-driven ‘Global Strategic Plan’ to achieve zero human deaths from dog-mediated rabies by 2030 [5]. This was recently given a massive boost when Gavi, the Vaccine Alliance, announced their decision to invest in human rabies vaccine for PEP [6]. While nation states are key to rabies elimination, as shown by examples from Latin America, South Africa, and Sri Lanka using existing knowledge, tools and technology [5], the importance of Gavi’s role cannot be understated. Unlike any other global or state actor, Gavi can directly address an otherwise intractable market failure in the supply of PEP. Market forces do not always serve LMICs well, where uncertain funding and demand for vaccines do not provide manufacturers with sufficient incentive to invest in products at affordable prices. Gavi has the power to ‘shape’ the market for human rabies vaccines.

In this commentary, we employ the Capabilities Approach to identify barriers to PEP access that lead to this market failure and show the role that Gavi can play in reducing exposure of PEP supply to market forces. The Capabilities Approach is concerned with what people are able to do and what lives they are able to lead. Only by assessing an individual’s capabilities can their real quality of life be determined. Applying the Approach is to identify the social arrangements that enable and impede people from fulfilling their capability [7,8]. People in LMICs have their capability to good health that is free from rabies constrained because of market and state failure to ensure PEP access. This is however not a concern for middle and high income countries. The Capabilities Approach is a normative theory of social justice that places a fundamental value on health and equity and advocates a fairer distribution of health capabilities. Traditional health policy making in contrast is largely dominated by a utilitarian approach, advocating social arrangements to maximise aggregate utility without directly taking account of distributional concerns [9]. This justifies large inequalities if an improvement in aggregate welfare is the end result [10]. This is the reason why rabies deaths occur mostly in LMICs and in the poorest communities even though these deaths are preventable through timely access to PEP. The Capabilities Approach provides a framework for including factors affecting an individual’s ability to access PEP in multiple and diverse contexts. In addition to the availability of PEP, it takes into account structural factors such as socioeconomic status, access to education, ability to travel or geographical location, country infrastructure, and other aspects of service provision that may have a role. In this way, the Capabilities Approach reveals the mechanisms by which inequity and injustice is manifested, which we outline below for PEP access to prevent human rabies.

Deep-rooted structural barriers across individual, national and global scales underpin the problem of limited PEP access. Strategies advocated by the World Bank, such as structural adjustment programmes and the introduction of user charges have widened health inequalities and inadvertently weakened service provision [11]. Bite victims in many LMICs face out-of-pocket costs of at least $10 per vaccine dose [12], and oftentimes over $100 for a multidose course [13], costs that are prohibitive for poor households. Health insurance schemes attempt to mitigate these costs, but only a small fraction of LMIC citizens have effective health insurance [14]. Moreover, these costs are compounded by travel; vaccines are usually only available in urban centres and in some countries only capital cities [12,13,15–19].

The lack of proper organization is also a key issue. Many LMICs still lack a national rabies programme [19]. At the same time, policies of decentralization have led many countries to devolve cost recovery responsibilities to local authorities. Much healthcare priority setting at the subnational level is often ad hoc [20]. Local budgets are usually allocated to cheaper medicines at the expense of less frequently used but, in the case of rabies, life-saving vaccines [12]. Thus without adequate PEP pre-financing (or aligned cost recovery), bite victims are either deferred to central hospitals where vaccines may be available [18], or there is a scramble to obtain vaccine from private providers [12,16]. Under these circumstances and a lack of regulatory oversight (transparent mechanisms accounting for vaccine use) the market price increases, leading to delays for patients and introducing a risk of PEP failure [4]. Inflated price for vaccines, including for non-pre-qualified vaccines of unassured quality [16], is left to the most vulnerable: bite victims and their families who are least able to pay, yet are facing a life or death situation [4]. Some suppliers are unwilling to provide vaccines if local authorities are indebted [12]. Insufficient procurement has been reported in several sub-Saharan countries to cause chronic stockouts [12,16,18]. The divergence of local health priorities from national priorities leads to individualized, sporadic and inadequate responses rather than a coherent population-level response. The reality is that PEP supply in many LMICs is not aligned with global recommendations or national strategies, and as such is neither functional nor responsive for those in need.

Global rabies policy has lacked coherence and translation into practice further constraining PEP access. A bewildering range of intramuscular and intradermal post-exposure regimens are considered safe and effective, each requiring different doses and clinic visits per course [21]. Intradermal vaccination uses considerably less vaccine per patient, enables more patients to be treated with the same vaccine volume (alleviating shortages), and is already routinely used for childhood BCG vaccination [21]. Yet, intradermal rabies post-exposure vaccination has not been widely adopted. Moreover, despite more than 30 years of evidence on the efficacy of intradermal post-exposure vaccination [22], pharmaceuticals have not added the route to their rabies vaccine labels. The complexity of regimens, minimal advertising of intradermal vaccination, and lack of simplified recommendations has contributed to inadequate PEP provision and confusion. Health workers in many LMICs are not aware of the intradermal option for rabies vaccination, with a lack of translation from international to local guidelines. Collectively, these barriers act as constraints on the ability to convert resources into functionings, known in the Capabilities Approach as ‘Conversion Factors’ [23]. Life-saving PEP exists but because of constrained access, the vulnerable lack real opportunity to health.

The latest WHO position on rabies creates an opportunity for harmonisation and global action [3]. The WHO now recommends a new dose sparing abridged intradermal regimen that uses just 0.6 mL of vaccine per course (less than all other regimens). It is completed in just one week [24]. Policies have been aligned to improve access to PEP [3]. The economic case is clear; adoption of WHO policies would be cost equivalent to the status quo [4]. Indeed current vaccine production could meet projected demand, reaching millions more people, through a switch to the recommended abridged intradermal regimen. But for social justice to be achieved and the WHO position to be realized, market shaping is required to overcome structural barriers and facilitate improved health seeking and adherence that would save many lives [4].

Making PEP free at point of care, as routine vaccines are, would immediately circumvent financial constraints on individuals and governments, and also reassure vaccine suppliers operating in LMICs. Beyond this, Gavi’s investment could bring health system benefits such as increased capacity for surveillance of human rabies exposures, deaths and accountable use of PEP that has been neglected for rabies. It also should create a translational opportunity for training healthcare workers to implement the new guidelines. Gavi investment has strengthened health systems and transparent supply chains for many vaccines, whereas systems for forecasting, procuring, distributing and monitoring rabies vaccines are mostly non-existent, inconsistent, and unresponsive [12,16,19]. Strengthened health systems improves equity and access to healthcare, and contributes towards long-term sustainable development. Bite victims will be able to overcome structural barriers to PEP access and fulfill their capability to health through avoidance of death from rabies.

The case for Gavi investment from a utilitarian cost-effectiveness perspective has been made elsewhere [4]. Using the Capabilities Approach, we make a normative argument by showing how the status quo of PEP supply is an unjust one. We have shown how those most at risk and in most need of rabies vaccines, face structural barriers which constrain their capabilities to a good health free from rabies. These barriers have also constrained their agency and political voice to advocate for change. Until recently, no powerful actors have led on policy change that could facilitate improved PEP access. Indeed, neither governments nor pharmaceuticals are incentivized to drive this change under current market forces. To overcome this persistent market failure in LMICs, Gavi’s investment could now redistribute the costs from those least able to pay, to global actors. This could also empower local social actors, from local communities to NGOs, to educate and help mobilise bite victims into seeking the care they need. This will change a currently unjust status quo, and help prevent the poorest from suffering disproportionately and dying unnecessarily from rabies.

While we recognise that the impact of global health initiatives play out in highly complex local realities, Gavi’s investment now potentially transforms a source of structural inequity in rabies prevention into an example of global health policy making that harnesses the potential that the SDGs offer [25]- an intervention that addresses both upstream and downstream causes of ill health. Improved PEP access should allow countries to redistribute resources within the health system. Moreover, drawing upon a collaborative One Health approach [26], we foresee more countries effectively leveraging on existing knowledge, tools and technology that others have already shown to be effective for rabies elimination [5]. This ought to catalyze mass dog vaccination programmes to eliminate rabies from source populations - the most equitable of solutions.
